# 1-Tetra­decyl­indoline-2,3-dione

**DOI:** 10.1107/S1600536810018258

**Published:** 2010-05-22

**Authors:** Khalil Mamari, Hafid Zouihri, El Mokhtar Essassi, Seik Weng Ng

**Affiliations:** a Laboratoire de Chimie Organique Hétérocyclique, Pôle de Compétences Pharmacochimie, Université Mohammed V-Agdal, BP 1014 Avenue Ibn Batout, Rabat, Morocco; b CNRST Division UATRS, Angle Allal Fassi/FAR, BP 8027 Hay Riad, Rabat, Morocco; c Department of Chemistry, University of Malaya, 50603 Kuala Lumpur, Malaysia

## Abstract

In the title *N*-alkyl isatin, C_22_H_33_NO_2_, the isatin moiety is planar (r.m.s. deviation = 0.03 Å). The tetra­decyl substituent has all torsion angles in an anti­periplanar conformation.

## Related literature

For background to *N*-substituted isatins and their derivatives, see: Bouhfid *et al.* (2008 ▶). For the crystal structures of two *N*-alkyl isatins, see: Miehe *et al.* (2003 ▶); Naumov *et al.* (2002 ▶).
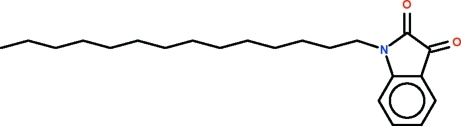


## Experimental

### 

#### Crystal data

C_22_H_33_NO_2_
*M* *_r_* = 343.49
Monoclinic, 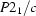

*a* = 27.6647 (8) Å
*b* = 4.7055 (1) Å
*c* = 15.7583 (5) Å
β = 103.635 (1)°
*V* = 1993.54 (10) Å^3^
*Z* = 4
Mo *K*α radiation
μ = 0.07 mm^−1^
*T* = 200 K
0.18 × 0.16 × 0.11 mm

#### Data collection

Bruker X8 APEXII diffractometer
23477 measured reflections
5172 independent reflections
3532 reflections with *I* > 2σ(*I*)
*R*_int_ = 0.042

#### Refinement

*R*[*F* ^2^ > 2σ(*F* ^2^)] = 0.065
*wR*(*F* ^2^) = 0.222
*S* = 1.11
5172 reflections
226 parameters
H-atom parameters constrained
Δρ_max_ = 0.27 e Å^−3^
Δρ_min_ = −0.21 e Å^−3^

### 

Data collection: *APEX2* (Bruker, 2008 ▶); cell refinement: *SAINT* (Bruker, 2008 ▶); data reduction: *SAINT*; program(s) used to solve structure: *SHELXS97* (Sheldrick, 2008 ▶); program(s) used to refine structure: *SHELXL97* (Sheldrick, 2008 ▶); molecular graphics: *X-SEED* (Barbour, 2001 ▶); software used to prepare material for publication: *publCIF* (Westrip, 2010 ▶).

## Supplementary Material

Crystal structure: contains datablocks global, I. DOI: 10.1107/S1600536810018258/bt5273sup1.cif

Structure factors: contains datablocks I. DOI: 10.1107/S1600536810018258/bt5273Isup2.hkl

Additional supplementary materials:  crystallographic information; 3D view; checkCIF report

## Figures and Tables

**Fig. 1.**
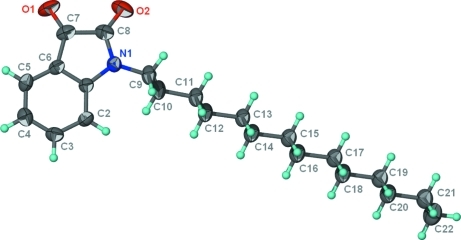
Anisotropic displacement ellipsoid plot (Barbour, 2001) of the title compound at the 70% probability level; hydrogen atoms are drawn as spheres of arbitrary radius.

## supplementary crystallographic information

### Comment

*N*-Substituted isatins (Bouhfid *et al.*, 2008) represent a large family of heterocyclic compounds reported to show a wide range of useful medicinal activities. These are readily synthesized by the reaction of isatin and an alkyl halide in the presence of a catalyst. The title tetradecyl derivative (Scheme I, Fig. 1) has a particarly long hydrocarbon chain; the chain adopts a extended zigzag conformation.

The crystal structures of only few *N*-substituted isatins have been reported; these have only short hydrocarbon chains, e.g., methyl isatin (Miehe *et al.*, 2003) and ethyl isatin (Naumov *et al.*, 2002).

### Experimental

To a solution of isatin (1 g, 6.8 mmol) dissolved in DMF(50 ml) was added 1-bromotetradecane (1.87 g, 6.8 mmol), potassium carbonate (1 g, 7.4 mmol) and a catalytic quantity of tetra-*n-*butylammonium bromide.The mixture was stirred for 48 h; the reaction was monitored by thin layer chromatography. The mixture was filtered and the solvent removed under vacuum. The solid that was obtained was recrystallized from ethanol to afford the title compound as orange crystals in 80% yield.

### Refinement

H-atoms were placed in calculated positions (C–H 0.95–0.99 Å) and were included in the refinement in the riding model approximation, with *U*(H) set to 1.2–1.5*U*_eq_(C).

### Crystal data

**Table:**

C_22_H_33_NO_2_	*F*(000) = 752
*M**_r_* = 343.49	*D*_x_ = 1.144 Mg m^−^^3^
Monoclinic, *P*2_1_/*c*	Mo *K*α radiation, λ = 0.71073 Å
Hall symbol: -P 2ybc	Cell parameters from 4926 reflections
*a* = 27.6647 (8) Å	θ = 2.6–28.7°
*b* = 4.7055 (1) Å	µ = 0.07 mm^−^^1^
*c* = 15.7583 (5) Å	*T* = 200 K
β = 103.635 (1)°	Irregular block, orange
*V* = 1993.54 (10) Å^3^	0.18 × 0.16 × 0.11 mm
*Z* = 4	

### Data collection

**Table:**

Bruker X8 APEXII diffractometer	3532 reflections with *I* > 2σ(*I*)
Radiation source: fine-focus sealed tube	*R*_int_ = 0.042
graphite	θ_max_ = 28.8°, θ_min_ = 2.3°
φ and ω scans	*h* = −37→37
23477 measured reflections	*k* = −6→5
5172 independent reflections	*l* = −21→21

### Refinement

**Table:**

Refinement on *F*^2^	Primary atom site location: structure-invariant direct methods
Least-squares matrix: full	Secondary atom site location: difference Fourier map
*R*[*F*^2^ > 2σ(*F*^2^)] = 0.065	Hydrogen site location: inferred from neighbouring sites
*wR*(*F*^2^) = 0.222	H-atom parameters constrained
*S* = 1.11	*w* = 1/[σ^2^(*F*_o_^2^) + (0.098*P*)^2^ + 1.1616*P*] where *P* = (*F*_o_^2^ + 2*F*_c_^2^)/3
5172 reflections	(Δ/σ)_max_ = 0.001
226 parameters	Δρ_max_ = 0.27 e Å^−^^3^
0 restraints	Δρ_min_ = −0.20 e Å^−^^3^

### Fractional atomic coordinates and isotropic or equivalent isotropic displacement parameters (Å^2^)

**Table:**

	*x*	*y*	*z*	*U*_iso_*/*U*_eq_	
O1	0.46046 (6)	1.1884 (4)	0.43051 (11)	0.0487 (5)	
O2	0.38053 (7)	0.7712 (5)	0.36601 (11)	0.0524 (5)	
N1	0.37452 (6)	0.7894 (4)	0.50944 (11)	0.0309 (4)	
C1	0.39707 (7)	0.9506 (4)	0.58407 (12)	0.0263 (4)	
C2	0.38674 (7)	0.9454 (5)	0.66525 (13)	0.0312 (4)	
H2	0.3623	0.8214	0.6780	0.037*	
C3	0.41379 (8)	1.1306 (5)	0.72815 (14)	0.0352 (5)	
H3	0.4073	1.1327	0.7847	0.042*	
C4	0.44969 (8)	1.3112 (5)	0.71059 (15)	0.0376 (5)	
H4	0.4672	1.4355	0.7548	0.045*	
C5	0.46031 (7)	1.3115 (5)	0.62847 (14)	0.0339 (5)	
H5	0.4852	1.4326	0.6160	0.041*	
C6	0.43356 (7)	1.1305 (4)	0.56570 (12)	0.0282 (4)	
C7	0.43501 (7)	1.0834 (5)	0.47474 (13)	0.0339 (5)	
C8	0.39388 (8)	0.8608 (5)	0.43985 (14)	0.0352 (5)	
C9	0.33228 (7)	0.5996 (5)	0.50282 (16)	0.0354 (5)	
H9A	0.3371	0.4853	0.5569	0.042*	
H9B	0.3306	0.4673	0.4534	0.042*	
C10	0.28326 (7)	0.7629 (5)	0.48906 (15)	0.0342 (5)	
H10A	0.2832	0.8755	0.5421	0.041*	
H10B	0.2808	0.8974	0.4399	0.041*	
C11	0.23799 (7)	0.5690 (5)	0.46968 (15)	0.0341 (5)	
H11A	0.2405	0.4329	0.5185	0.041*	
H11B	0.2377	0.4583	0.4161	0.041*	
C12	0.18917 (7)	0.7341 (5)	0.45715 (15)	0.0346 (5)	
H12A	0.1886	0.8336	0.5123	0.042*	
H12B	0.1880	0.8803	0.4115	0.042*	
C13	0.14296 (7)	0.5478 (5)	0.43108 (15)	0.0347 (5)	
H13A	0.1440	0.4011	0.4765	0.042*	
H13B	0.1433	0.4492	0.3757	0.042*	
C14	0.09459 (7)	0.7159 (5)	0.41929 (15)	0.0359 (5)	
H14A	0.0941	0.8117	0.4750	0.043*	
H14B	0.0939	0.8651	0.3748	0.043*	
C15	0.04810 (7)	0.5345 (5)	0.39165 (15)	0.0364 (5)	
H15A	0.0487	0.3850	0.4361	0.044*	
H15B	0.0485	0.4391	0.3358	0.044*	
C16	0.00019 (7)	0.7038 (5)	0.38033 (15)	0.0373 (5)	
H16A	−0.0003	0.8538	0.3361	0.045*	
H16B	−0.0001	0.7987	0.4363	0.045*	
C17	−0.04666 (7)	0.5241 (5)	0.35234 (15)	0.0376 (5)	
H17A	−0.0463	0.3745	0.3967	0.045*	
H17B	−0.0463	0.4288	0.2965	0.045*	
C18	−0.09460 (7)	0.6944 (5)	0.34082 (16)	0.0377 (5)	
H18A	−0.0948	0.7913	0.3965	0.045*	
H18B	−0.0951	0.8430	0.2961	0.045*	
C19	−0.14159 (7)	0.5157 (6)	0.31364 (16)	0.0386 (5)	
H19A	−0.1415	0.4188	0.2579	0.046*	
H19B	−0.1411	0.3672	0.3583	0.046*	
C20	−0.18930 (8)	0.6862 (6)	0.30219 (16)	0.0390 (5)	
H20A	−0.1894	0.7831	0.3579	0.047*	
H20B	−0.1897	0.8348	0.2575	0.047*	
C21	−0.23622 (8)	0.5095 (6)	0.27508 (17)	0.0447 (6)	
H21A	−0.2358	0.3606	0.3196	0.054*	
H21B	−0.2363	0.4133	0.2192	0.054*	
C22	−0.28379 (8)	0.6818 (7)	0.2641 (2)	0.0543 (7)	
H22A	−0.3126	0.5554	0.2469	0.081*	
H22B	−0.2850	0.8263	0.2188	0.081*	
H22C	−0.2844	0.7747	0.3195	0.081*	

### Atomic displacement parameters (Å^2^)

**Table:**

	*U*^11^	*U*^22^	*U*^33^	*U*^12^	*U*^13^	*U*^23^
O1	0.0391 (9)	0.0730 (13)	0.0381 (9)	0.0013 (9)	0.0171 (7)	0.0179 (9)
O2	0.0500 (10)	0.0715 (13)	0.0335 (9)	0.0075 (10)	0.0056 (7)	−0.0139 (8)
N1	0.0226 (8)	0.0361 (10)	0.0329 (9)	−0.0005 (7)	0.0045 (7)	−0.0041 (7)
C1	0.0209 (8)	0.0309 (10)	0.0261 (9)	0.0024 (8)	0.0035 (7)	0.0019 (8)
C2	0.0268 (9)	0.0377 (11)	0.0301 (10)	−0.0018 (9)	0.0087 (8)	0.0045 (8)
C3	0.0351 (11)	0.0438 (13)	0.0269 (10)	0.0039 (10)	0.0078 (8)	0.0003 (9)
C4	0.0347 (11)	0.0366 (12)	0.0382 (11)	−0.0014 (10)	0.0023 (9)	−0.0041 (9)
C5	0.0246 (9)	0.0355 (11)	0.0398 (11)	−0.0036 (9)	0.0041 (8)	0.0047 (9)
C6	0.0222 (9)	0.0337 (11)	0.0287 (9)	0.0030 (8)	0.0060 (7)	0.0067 (8)
C7	0.0256 (9)	0.0451 (13)	0.0314 (10)	0.0094 (9)	0.0073 (8)	0.0121 (9)
C8	0.0297 (10)	0.0450 (13)	0.0301 (10)	0.0104 (10)	0.0055 (8)	−0.0007 (9)
C9	0.0223 (9)	0.0332 (11)	0.0478 (12)	−0.0004 (9)	0.0026 (8)	−0.0053 (9)
C10	0.0227 (9)	0.0297 (11)	0.0475 (12)	−0.0006 (8)	0.0030 (9)	−0.0047 (9)
C11	0.0208 (9)	0.0342 (11)	0.0450 (12)	0.0001 (8)	0.0032 (8)	−0.0041 (9)
C12	0.0217 (9)	0.0344 (11)	0.0457 (12)	0.0001 (8)	0.0036 (8)	−0.0026 (9)
C13	0.0208 (9)	0.0390 (12)	0.0423 (11)	−0.0007 (9)	0.0035 (8)	−0.0028 (9)
C14	0.0218 (9)	0.0404 (12)	0.0443 (12)	0.0005 (9)	0.0056 (8)	−0.0012 (10)
C15	0.0220 (9)	0.0432 (13)	0.0425 (12)	−0.0004 (9)	0.0044 (9)	−0.0026 (10)
C16	0.0225 (9)	0.0424 (13)	0.0456 (12)	0.0003 (9)	0.0055 (9)	−0.0017 (10)
C17	0.0225 (9)	0.0447 (13)	0.0439 (12)	−0.0001 (9)	0.0042 (9)	−0.0038 (10)
C18	0.0211 (9)	0.0437 (13)	0.0460 (12)	0.0001 (9)	0.0032 (9)	−0.0012 (10)
C19	0.0228 (9)	0.0474 (14)	0.0442 (12)	0.0003 (9)	0.0050 (9)	−0.0048 (10)
C20	0.0237 (10)	0.0454 (13)	0.0458 (12)	0.0004 (10)	0.0042 (9)	−0.0012 (10)
C21	0.0277 (11)	0.0535 (15)	0.0508 (14)	−0.0028 (11)	0.0052 (10)	−0.0085 (12)
C22	0.0246 (11)	0.0702 (19)	0.0649 (17)	−0.0010 (12)	0.0045 (11)	−0.0017 (15)

### Geometric parameters (Å, °)

**Table:**

O1—C7	1.207 (2)	C13—H13A	0.9900
O2—C8	1.211 (3)	C13—H13B	0.9900
N1—C8	1.371 (3)	C14—C15	1.519 (3)
N1—C1	1.415 (3)	C14—H14A	0.9900
N1—C9	1.455 (3)	C14—H14B	0.9900
C1—C2	1.375 (3)	C15—C16	1.520 (3)
C1—C6	1.399 (3)	C15—H15A	0.9900
C2—C3	1.397 (3)	C15—H15B	0.9900
C2—H2	0.9500	C16—C17	1.523 (3)
C3—C4	1.384 (3)	C16—H16A	0.9900
C3—H3	0.9500	C16—H16B	0.9900
C4—C5	1.393 (3)	C17—C18	1.523 (3)
C4—H4	0.9500	C17—H17A	0.9900
C5—C6	1.381 (3)	C17—H17B	0.9900
C5—H5	0.9500	C18—C19	1.522 (3)
C6—C7	1.460 (3)	C18—H18A	0.9900
C7—C8	1.549 (3)	C18—H18B	0.9900
C9—C10	1.529 (3)	C19—C20	1.519 (3)
C9—H9A	0.9900	C19—H19A	0.9900
C9—H9B	0.9900	C19—H19B	0.9900
C10—C11	1.521 (3)	C20—C21	1.516 (3)
C10—H10A	0.9900	C20—H20A	0.9900
C10—H10B	0.9900	C20—H20B	0.9900
C11—C12	1.530 (3)	C21—C22	1.520 (3)
C11—H11A	0.9900	C21—H21A	0.9900
C11—H11B	0.9900	C21—H21B	0.9900
C12—C13	1.524 (3)	C22—H22A	0.9800
C12—H12A	0.9900	C22—H22B	0.9800
C12—H12B	0.9900	C22—H22C	0.9800
C13—C14	1.527 (3)		
			
C8—N1—C1	110.78 (17)	H13A—C13—H13B	107.8
C8—N1—C9	123.48 (18)	C15—C14—C13	113.71 (19)
C1—N1—C9	125.31 (17)	C15—C14—H14A	108.8
C2—C1—C6	121.43 (19)	C13—C14—H14A	108.8
C2—C1—N1	128.04 (18)	C15—C14—H14B	108.8
C6—C1—N1	110.53 (16)	C13—C14—H14B	108.8
C1—C2—C3	117.06 (19)	H14A—C14—H14B	107.7
C1—C2—H2	121.5	C14—C15—C16	113.3 (2)
C3—C2—H2	121.5	C14—C15—H15A	108.9
C4—C3—C2	122.05 (19)	C16—C15—H15A	108.9
C4—C3—H3	119.0	C14—C15—H15B	108.9
C2—C3—H3	119.0	C16—C15—H15B	108.9
C3—C4—C5	120.3 (2)	H15A—C15—H15B	107.7
C3—C4—H4	119.8	C15—C16—C17	113.7 (2)
C5—C4—H4	119.8	C15—C16—H16A	108.8
C6—C5—C4	118.04 (19)	C17—C16—H16A	108.8
C6—C5—H5	121.0	C15—C16—H16B	108.8
C4—C5—H5	121.0	C17—C16—H16B	108.8
C5—C6—C1	121.07 (18)	H16A—C16—H16B	107.7
C5—C6—C7	131.52 (19)	C16—C17—C18	113.6 (2)
C1—C6—C7	107.41 (18)	C16—C17—H17A	108.8
O1—C7—C6	131.3 (2)	C18—C17—H17A	108.8
O1—C7—C8	123.5 (2)	C16—C17—H17B	108.8
C6—C7—C8	105.27 (16)	C18—C17—H17B	108.8
O2—C8—N1	126.5 (2)	H17A—C17—H17B	107.7
O2—C8—C7	127.5 (2)	C19—C18—C17	113.9 (2)
N1—C8—C7	105.93 (17)	C19—C18—H18A	108.8
N1—C9—C10	111.84 (18)	C17—C18—H18A	108.8
N1—C9—H9A	109.2	C19—C18—H18B	108.8
C10—C9—H9A	109.2	C17—C18—H18B	108.8
N1—C9—H9B	109.2	H18A—C18—H18B	107.7
C10—C9—H9B	109.2	C20—C19—C18	113.7 (2)
H9A—C9—H9B	107.9	C20—C19—H19A	108.8
C11—C10—C9	112.80 (18)	C18—C19—H19A	108.8
C11—C10—H10A	109.0	C20—C19—H19B	108.8
C9—C10—H10A	109.0	C18—C19—H19B	108.8
C11—C10—H10B	109.0	H19A—C19—H19B	107.7
C9—C10—H10B	109.0	C21—C20—C19	114.0 (2)
H10A—C10—H10B	107.8	C21—C20—H20A	108.8
C10—C11—C12	112.39 (18)	C19—C20—H20A	108.8
C10—C11—H11A	109.1	C21—C20—H20B	108.8
C12—C11—H11A	109.1	C19—C20—H20B	108.8
C10—C11—H11B	109.1	H20A—C20—H20B	107.7
C12—C11—H11B	109.1	C20—C21—C22	113.6 (2)
H11A—C11—H11B	107.9	C20—C21—H21A	108.8
C13—C12—C11	113.66 (19)	C22—C21—H21A	108.8
C13—C12—H12A	108.8	C20—C21—H21B	108.8
C11—C12—H12A	108.8	C22—C21—H21B	108.8
C13—C12—H12B	108.8	H21A—C21—H21B	107.7
C11—C12—H12B	108.8	C21—C22—H22A	109.5
H12A—C12—H12B	107.7	C21—C22—H22B	109.5
C12—C13—C14	112.95 (19)	H22A—C22—H22B	109.5
C12—C13—H13A	109.0	C21—C22—H22C	109.5
C14—C13—H13A	109.0	H22A—C22—H22C	109.5
C12—C13—H13B	109.0	H22B—C22—H22C	109.5
C14—C13—H13B	109.0		
			
C8—N1—C1—C2	178.1 (2)	C1—N1—C8—C7	2.6 (2)
C9—N1—C1—C2	5.4 (3)	C9—N1—C8—C7	175.52 (18)
C8—N1—C1—C6	−1.6 (2)	O1—C7—C8—O2	−2.9 (4)
C9—N1—C1—C6	−174.36 (18)	C6—C7—C8—O2	175.9 (2)
C6—C1—C2—C3	0.9 (3)	O1—C7—C8—N1	178.6 (2)
N1—C1—C2—C3	−178.8 (2)	C6—C7—C8—N1	−2.7 (2)
C1—C2—C3—C4	−0.4 (3)	C8—N1—C9—C10	−94.4 (2)
C2—C3—C4—C5	−0.5 (3)	C1—N1—C9—C10	77.5 (3)
C3—C4—C5—C6	0.9 (3)	N1—C9—C10—C11	172.23 (18)
C4—C5—C6—C1	−0.4 (3)	C9—C10—C11—C12	179.29 (19)
C4—C5—C6—C7	178.9 (2)	C10—C11—C12—C13	175.77 (19)
C2—C1—C6—C5	−0.5 (3)	C11—C12—C13—C14	179.72 (19)
N1—C1—C6—C5	179.22 (19)	C12—C13—C14—C15	178.93 (19)
C2—C1—C6—C7	−179.99 (19)	C13—C14—C15—C16	179.85 (19)
N1—C1—C6—C7	−0.3 (2)	C14—C15—C16—C17	179.82 (19)
C5—C6—C7—O1	1.0 (4)	C15—C16—C17—C18	−179.84 (19)
C1—C6—C7—O1	−179.6 (2)	C16—C17—C18—C19	−179.47 (19)
C5—C6—C7—C8	−177.6 (2)	C17—C18—C19—C20	−179.98 (19)
C1—C6—C7—C8	1.8 (2)	C18—C19—C20—C21	180.0 (2)
C1—N1—C8—O2	−176.0 (2)	C19—C20—C21—C22	179.8 (2)
C9—N1—C8—O2	−3.1 (3)		
